# Investigation of temperature-dependent photoluminescence in multi-quantum wells

**DOI:** 10.1038/srep12718

**Published:** 2015-07-31

**Authors:** Yutao Fang, Lu Wang, Qingling Sun, Taiping Lu, Zhen Deng, Ziguang Ma, Yang Jiang, Haiqiang Jia, Wenxin Wang, Junming Zhou, Hong Chen

**Affiliations:** 1 Key Laboratory for Renewable Energy, Chinese Academy of Sciences, China; 2 Beijing Key Laboratory for New Energy Materials and Devices, Beijing National Laboratory for Condensed Matter Physics, Institute of Physics, Chinese Academy of Sciences, Beijing 100190, China

## Abstract

Photoluminescence (PL) is a nondestructive and powerful method to investigate carrier recombination and transport characteristics in semiconductor materials. In this study, the temperature dependences of photoluminescence of GaAs-Al_x_Ga_1-x_As multi-quantum wells samples with and without p-n junction were measured under both resonant and non-resonant excitation modes. An obvious increase of photoluminescence(PL) intensity as the rising of temperature in low temperature range (T < 50 K), is observed only for GaAs-Al_x_Ga_1-x_As quantum wells sample with p-n junction under non-resonant excitation. The origin of the anomalous increase of integrated PL intensity proved to be associated with the enhancement of carrier drifting because of the increase of carrier mobility in the temperature range from 15 K to 100 K. For non-resonant excitation, carriers supplied from the barriers will influence the temperature dependence of integrated PL intensity of quantum wells, which makes the traditional methods to acquire photoluminescence characters from the temperature dependence of integrated PL intensity unavailable. For resonant excitation, carriers are generated only in the wells and the temperature dependence of integrated PL intensity is very suitable to analysis the photoluminescence characters of quantum wells.

Photoluminescence (PL) technique, a nondestructive and powerful method , has been widely used for the investigation of optical and electrical characters of semiconductors, such as carriers lifetime^1^,^2^, luminescence of impurities^3^,^4^ and band structures^5^,^6^,^7^. Moreover, the PL technique is also an effective method for investigating the novel characters of low-dimensional structure, such as quantum wells^8^,^9^,^10^, quantum wires^11^,^12^,^13^ and quantum dots^14^,^15^ and an useful tool for exploring new materials for optical and electrical devices^16^,^17^. Because of the importance of the PL technique, it is crucial to get a deeper understanding of the PL process, especially the transport of photogenerated carriers in the PL process. Resonant and non-resonant excitation modes are two widely used PL methods, and different carrier transport processes may occur under above excitation conditions^18^,^19^. Therefore, PL measurements under different excitation modes may reflect different aspects of the studied materials^20^, and the application ranges of the different excitation modes are needed to be further clarified.

In our previous paper^20^, we have shown that resonant excitation PL is suitable for investing the intrinsic PL characters of the InGaN quantum wells, while non-resonant excitation PL provides certain advantages for investigating the carrier transport dynamics and evaluating the internal quantum efficiency. However, because of the intrinsic large polarization field existing in the InGaN/GaN quantum wells, this structure is not an ideal structure for distinguishing the different effects of carrier drifting and carrier diffusion on the PL characters under non-resonant excitation mode. In order to further illustrate the carrier transport mechanism in the PL process under non-resonant excitation, GaAs/AlGaAs multiple quantum well structure is introduced. One advantage of GaAs/AlGaAs structure is that high-quality AlGaAs/GaAs quantum wells can be grown either by molecular-beam epitaxy (MBE ) or by metal-organic chemical vapour deposition^21^,^22^, because of the low lattice mismatch between AlAs and GaAs. The other more important advantage is that there is no polarization field and inhomogeneous structures in GaAs quantum wells grown on GaAs (001) substrates^23^. Due to the above advantages of GaAs/AlGaAs quantum wells, the specific roles of drifting and diffusion played in the PL process under non-resonant excitation can be distinguished in this structure.

In this study, the GaAs/AlGaAs multi-quantum well structure is used to clarify the specific origin of the abnormal increase of PL intensity and the application range of different excitation modes. Two samples of GaAs/AlGaAs multi-quantum wells(MQWs) with and without the p-n junction, grown on (001) GaAs substrates by MBE are used as the particular samples for the investigation of carrier transport mechanism in the PL process. An increase of integrated PL intensity as the rising of temperature is observed only for the sample with the p-n junction under non-resonant excitation mode, while for the sample without p-n junction or under resonant excitation mode, such an anomalous PL character is not observed. Because of the temperature-dependent carrier supply from the barriers to wells under resonant excitation, the temperature-dependent integrated PL intensity is not suitable to acquire the characters of nonradiative characters of GaAs wells. For PL measured under resonant excitation, carriers are generated only in the wells and the temperature-dependent integrated PL intensity can reflect the characters of nonradiative channel in the GaAs wells. A modified Arrhenius formula taking into account the temperature-dependent of radiative lifetime is proposed to fit the integrated PL intensities in this study. The specific origin of the anomalous temperature dependence of photoluminescence is clarified and nonradiative characters of GaAs quantum wells are investigated using a modified Arrhenius equation under resonant excitation.

## Results

### Photoluminescence mechanism under resonant and non-resonant excitation

**Figure 1:**
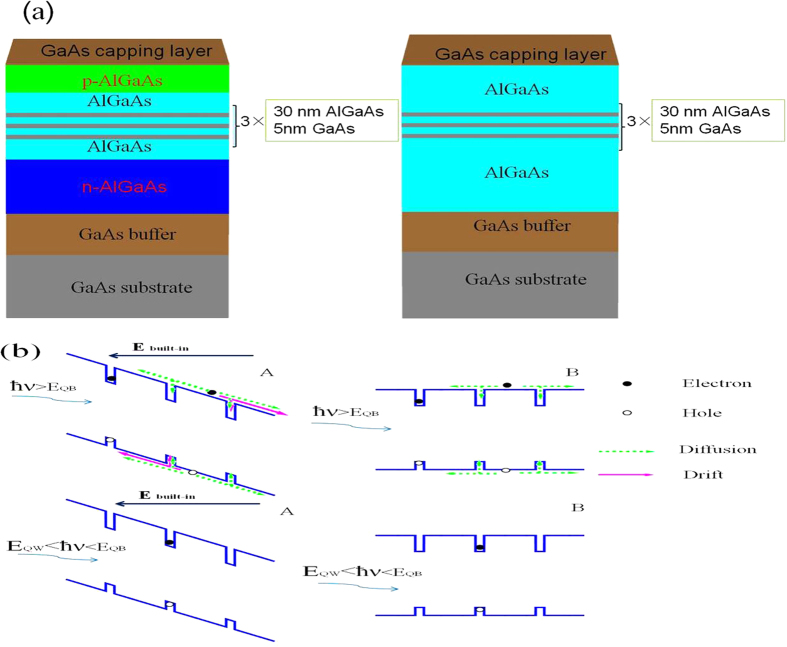
Multi-quantum well structures with and without p-n junction. (**a**) The structure diagram of sample A with p-n junction and sample B without p-n junction. (**b**)Schematic of carrier transport in the PL process for sample A and sample B taking into account the non-resonant excitation(ħν > E_QB_) and resonant excitation(E_QW_ < ħν < E_QB_). Carrier transport from barriers to wells occurs only under non-resonant excitation mode and both carrier drifting and diffusion occur in sample A while only diffusion occurs in sample B. E_QB_ is the energy gap of barriers, E_QW_ is the energy gap of wells and ħν is the energy of excitation photon.

The schematic diagram of sample structures is shown in Fig. 1(a) for sample A and sample B. The only difference between the two samples is that sample A has a n-doped AlGaAs layer below the quantum wells and a p-doped AlGaAs layer above the quantum wells, while sample B has two undoped AlGaAs layers. Therefore, sample A is the sample with p-n junction and sample B is the one without p-n junction. The energy band structures of sample A and sample B are shown schematically in Fig. 1(b), along the growth direction. As shown in Fig. 1(b), carrier transport from barriers to wells occurs when non-resonant excitation is used. Diffusion and drifting are two main mechanisms, which may account for the transport of carriers in PL process. The carrier concentration gradient is the driving force for diffusion and the electric field is the driving force for drifting. For sample A, because of the existing of built-in electric field, carriers transport from barriers to wells can be both diffusion and drifting, while for sample B, carriers generated in barriers can transport to wells only through diffusion under non-resonant excitation. Under resonant excitation, carriers are only generated in the wells and the barriers are not optically excited because the energy of excitation photon is lower than the energy of barrier band gap. Hence, no carriers transport from barriers to wells will happen under the resonant excitation mode. Consequently, the temperature dependence of integrated PL intensity measured under resonant excitation mode can reflect the thermal activation characters of non-radiative combination channels in the quantum wells. However, under the non-resonant excitation, temperature dependent supply of carriers from barriers through diffusion and drift will influence the PL intensity of quantum wells. As a result, the temperature dependence of integrated PL intensity measured under non-resonant excitation reflects the combination effects of carrier transport from barriers to wells and carriers recombination in the wells, which can not be used to analysis the non-radiative mechanism in the quantum wells by the traditional Arrhenius plot.

### Temperature-dependent integrated photoluminescence intensities measured under non-resonant excitation modes

**Figure 2:**
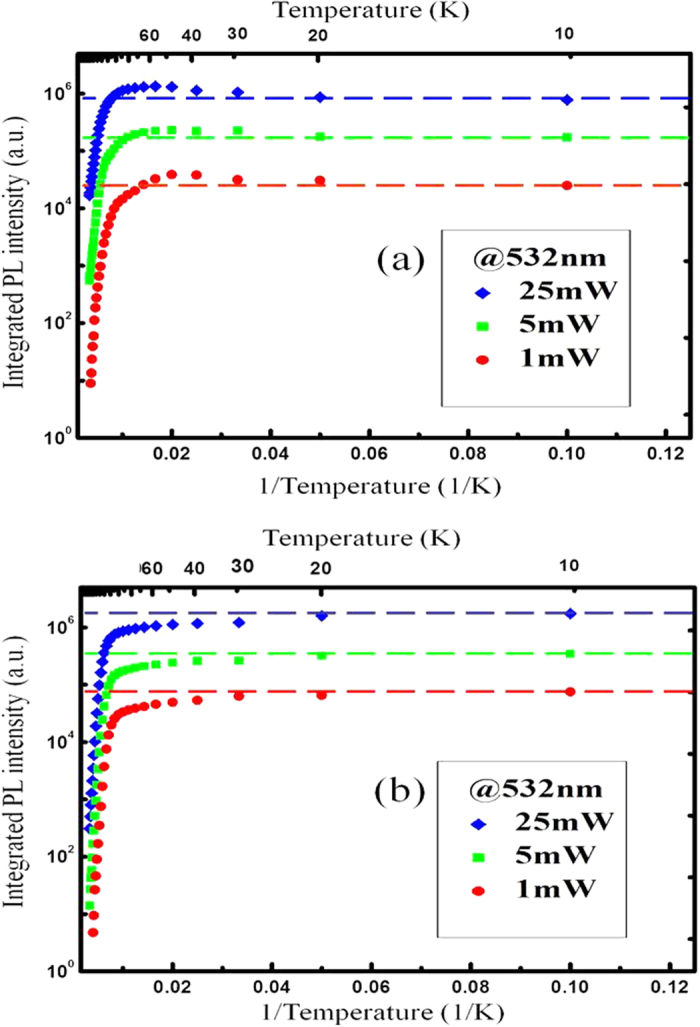
Temperature-dependent integrated PL intensities of GaAs quantum wells obtained by non-resonant excitation under various powers. The dashed lines represent the intensities equal to the intensities at 10 K for each excitation powers. (**a**) The temperature-dependent integrated PL intensities for Sample A with p-n junction under non-resonant excitation. The anomalous increase of integrated PL intensity are observed in the low temperature range (below 50 K) under different excitation powers. (**b**) The temperature-dependent integrated PL intensities for Sample B without p-n junction under non-resonant excitation. The monotonous decrease of the integrated PL intensities are observed in whole measured temperature range under different excitation powers.

Figure 2 shows the temperature dependences of integrated PL intensity obtained by non-resonant excitation under varied powers for sample A and sample B. The energy of excitation photon is 2.33 eV, above the band gap of Al_0.28_Ga_0.72_As barrier, so the barriers are optically excited. As a result, there is a temperature dependent carrier supply from barriers to wells under non-resonant excitation mode. It is clearly revealed in Fig. 2 that the integrated PL intensities show a gradually increase for the sample A as the temperature increased from 10 K to about 50 K, while for the sample B such an increase is not observed. This anomalous phenomenon can be interpreted as the increase of carriers captured into the wells from the barriers as rising of temperature in low temperature range. Drifting and diffusion are the two possible ways of carrier transport from the barriers into wells. Diffusion exists in both samples under the driving force of carrier concentration gradient and drifting occurs only in sample A under the driving force of built-in field. From the comparison of sample A and B, we can conclude that the enhancement of carrier drifting from barriers is the major contribution to the exceptional increase of PL intensities. Because of the low activation energies of the Si and Be dopants in AlGaAs layer^24^, the temperature dependence of built-in electric field can be neglected in the temperature range of measurement. The low-temperature carrier mobility is governed by ionized-impurity scattering and space-charge scattering and shows a monotonous increase in the low temperature range (~15 K-100 K)^25^,^26^. This increasing of carrier mobility will enhance the carrier drifting under the built-in filed and the amount of carriers captured by the GaAs wells is increase as rising of temperature in the low temperature range. As a consequence, the increasing of PL intensity is observed in the low temperature range for the sample A, while there is no observable abnormal increase of PL intensity for the sample B. There is no built-in electronic field in sample B, so the carrier transport from the barriers to wells is diffusion. Therefore, the mechanism responsible for the anomalous increase of PL intensity is the carrier drifting, not the carrier diffusion in our experiments.

For sample A, the enhancement of carriers captured by the wells and decreasing of internal quantum efficiency as the rising of temperature result in a peak of PL intensity at the temperature around 50 K. Moreover, as the increase of excitation power, the temperature for the onset of PL intensity decrease moves to higher temperature, shown in Fig. 2 (a). The shift of the peak temperature of integrated PL intensity may be attribute to the increase of the carrier lifetime in the barriers when increasing the excitation power, because of the saturation of recombination centers^27^. When the lifetime of carriers in the barriers increase, more carriers will be captured into the wells, which will compensate the decreasing of radiative recombination efficiency at higher temperature. As a result, the inflection temperature of PL intensity moves to higher temperature as the increasing of excitation power, shown in Fig. 2(a). From the non-resonant excitation measurement results, we can conclude that the enhancement of carrier drifting as the rising of temperature is caused by the increasing of carrier mobility and is responsible for the abnormal increase of PL intensity for sample A.

### Temperature-dependent integrated PL intensities measured under resonant excitation modes

**Figure 3:**
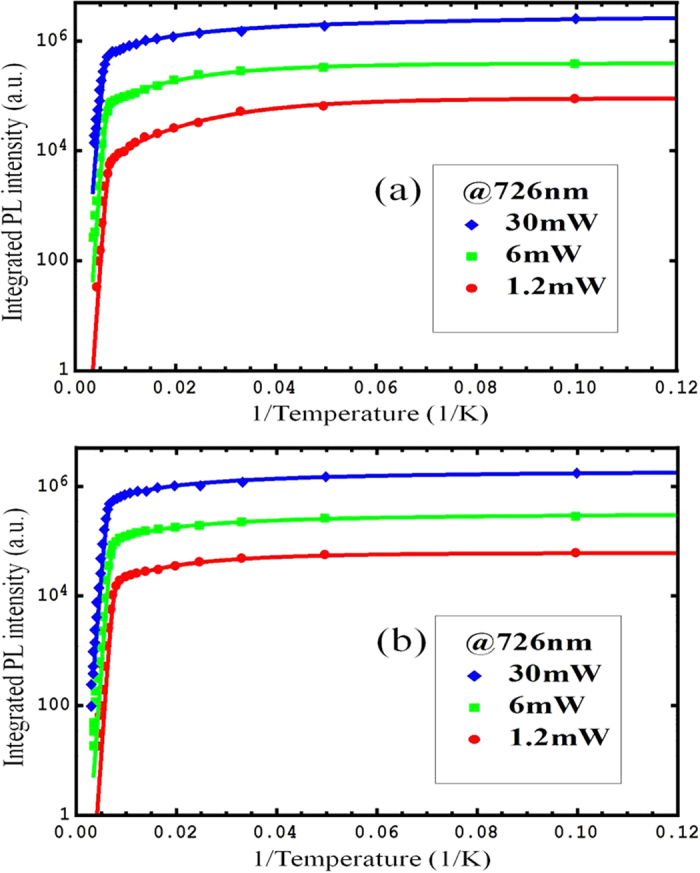
Temperature-dependent integrated PL intensities of GaAs quantum wells obtained by resonant excitation under various powers. The solid curves represent the best fits for the PL intensities as a function of temperature, using the modified Arrhenius formula described in the text. (**a**) The temperature-dependent integrated PL intensities for Sample A with p-n junction under resonant excitation. (**b**) The temperature-dependent integrated PL intensities for Sample B without p-n junction under non-resonant excitation.

In order to further illustrate the cause of the anomalous increase of PL intensities, we measured the temperature-dependent photoluminescence of sample A and sample B under resonant excitation with the excited energy of 1.71 eV below the band gap of Al_0.28_Ga_0.72_As barrier. Under resonant excitation, carriers are generated only in the wells and the effects of carrier supply from the barriers can be eliminated. The temperature-dependent integrated PL intensities of sample A and sample B are shown in Fig. 3, obtained by resonant excitation with varied excitation powers. The exceptional increase of PL intensities is not observed in Fig. 3 for both of sample A and sample B, which further proves that the supply of photogenerated carriers from barriers to GaAs wells under non-resonant excitation is the cause of the anomalous increase of integrated PL intensity.

To clarify the mechanism of PL quenching as the rising of temperature, the temperature-dependent integrated PL intensities measured under resonant excitation are fitted using a modified Arrhenius formula. Typically, for one nonradiative recombination channel, the Arrhenius formula is given by^28^.


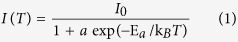


where *I*(T) represents the integrated PL intensity at temperature of T. The parameter *I*_0_ is the integrated PL intensity when the temperature approaching 0 K. E_*a*_ is the activation energy of nonradiative channel and *k*_*B*_ is the Boltzmann’s constant. The parameter *a* is obtained by


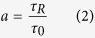


where τ_*R*_ is radiative lifetime of carriers and τ_0_ is the constant related to nonradiative lifetime of carriers. Traditionally the temperature dependence of τ_*R*_ is neglected in the Arrhenius formula and the parameter a is treated as a constant^28^,^29^. However, for the GaAs quantum well the radiative lifetime of carriers approximately linearly depends on temperature^30^, so parameter *a* can not be treated as constant. Consequently, the Arrhenius formula can be revised as


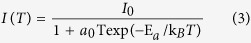


with 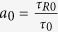
and 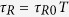. In this paper, we propose that there are two nonradiative recombination channels related to the quenching mechanism of PL intensities for the GaAs quantum wells. Then the modified Arrhenius formula used to fit our experiment data can be written as


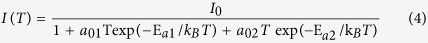


where *a*_01_ and E_*a*1_ are related to the nonradiative recombination channel in the GaAs wells and *a*_02_ and E_*a*2_ are related to the nonradiative recombination channel in Al_x_Ga_1-x_As barriers^31^. Unlike the traditional Arrhenius plot fitting, our modified Arrhenius formula can be used to fit the integrated PL intensities very well in the whole measured temperature range, as the solid line shown in Fig. 3. All fitting results for both samples under different powers, are listed in Table 1. As the increase of photogenerated carrier density, the exciton binding energy decreases because of the enhancement of carrier screening effect. Therefore, the activation energies for thermal quenching of the exciton luminescence decrease at high excitation power, which is consistent with the fitting results of activation energies, shown in Table 1. Moreover the decreases of *a*_01_ and *a*_02_ as the increase of excitation power can be interpreted as the consequence of increase of τ_0_. Because of the saturation of the nonradiative centers, the constant τ_0_ increases as the increasing of excitation power which in turn leads to the decrease of *a*_01_ and *a*_02_. The differences of the coefficients *a*_01_,* a*_02_ between sample A and sample B, are proposed to be attribute to the increase of carrier life time under electrical field^32^,^33^. The increase of radiative lifetime dominates for *a*_01_, leading to a larger *a*_01_ of sample A, while the increase of non-radiative lifetime dominates for *a*_02_, resulting in a smaller *a*_02_ of sample A. The fitting activation energies of E_*a*2_ are around 220 m eV, which are comparable to the barrier height of the exciton in the quantum wells. These fitting results further prove that the photoluminescence quenching at high temperature is caused by the nonradiative recombination of carriers escaped from the well to barrier, which is consistent with previously reported results^34^,^35^.

## Discussion

The PL characters of GaAs-AlGaAs MQWs were measured for the samples with and without p-n junction under both non-resonant and resonant excitation. The exceptional increase of integrate PL intensities as rising of temperature, is found to be mainly caused by the drifting of photogenerated carriers from barriers to wells under non-resonant excited. The increasing of carrier mobility in the low temperature (~15 K-100 K) is believed to be the specific cause of the anomalous temperature dependence PL intensity. The temperature-dependent integrated PL intensities under resonant excitation can be well fitted by the modified Arrhenius formula with two nonradiative recombination channels in the whole measured temperature. The activation energies of PL thermal quenching at high temperature fitted by the modified Arrhenius formula are compare to the barrier height for the exciton escaping out of well, which confirms that the non-radiative channel at high temperature is related to the non-radiative centers in barriers.

Because of the superiority of GaAs/AlGaAs structure, we are able to distinguish the roles of carrier diffusion and drifting played in PL process of quantum wells measured under non-resonant excitation. The origin of the increase of PL intensity as the rising of temperature is elucidated and the application range of PL measured under different excitation modes are confirmed. The resonant excitation mode is suitable to investigate the luminescence characters of quantum wells, while the non-resonant excitation mode reflects a combining effect of carrier transport and carrier recombination and is not a proper way to investigate the optical characters of quantum wells directly. The modified Arrhenius formula taking into account the temperature dependence of radiative lifetime of quantum well has been used to fit the temperature dependent integrate PL intensities under the resonant excitation. The results acquired in this work will help to get a comprehensive picture of PL process and serve as a guiding role for the PL measurement of low dimensional semiconductor structures.

## Methods

### Sample growth

The samples studied in this paper are grown on the semi-insulating (001) GaAs substrate at 580 °C by VG-80H solid-source MBE system in which solid elements (Ga, Al, As, Si and Be) are used as sources. The epitaxial films were grown in a As_2_-rich condition with a valved arsenic cracker cell at temperature of 850 °C. For the sample A, firstly, a 400 nm GaAs layer was grown on GaAs (001) surface followed by a 300 nm Si-doped AlGaAs layer and a 130 nm undoped AlGaAs layer . Then 3 periods GaAs/AlGaAs MQWs with 5 nmGaAs Wells separated by 30 nm AlGaAs barriers were grown. Finally, a 100 nm AlGaAs layer and a 150 nm Be-doped AlGaAs layer were grown on the MQW with a 8 nm GaAs capping layer on the top. Sample B has the same grown structure as sample A except that there is no dopant used during the growth of AlGaAs layers for sample B.

### Photoluminescence measurements

Temperature-dependent PL measurements are performed with the sample mounted in a closed-loop He gas cryostat, providing the measurement temperature range from 10 K to 300 K. A 532 nm semiconductor laser diode is used as the non-resonant excitation and a 726 nm semiconductor laser diode is used as resonant excitation. The size of the light spot focused on the samples was approximately 0.1 mm. The excitation power using in this paper are adjusted by a neutral optical attenuator. The PL signal is analysis with a triple-grating 50-cm monochromator and detector by Si photomultiplier tube using the conventional lock-in technique.

## Additional Information

**How to cite this article**: Fang, Y. *et al.* Investigation of temperature-dependent photoluminescence in multi-quantum wells. *Sci. Rep.* **5**, 12718; doi: 10.1038/srep12718 (2015).

## Figures and Tables

**Table 1:** The obtained fitting parameters: activation energies (E_*a*1_ and E_*a*2_) and constants (*a*_01_ and *a*_02_).

Excitation power	**726 nm laser-sample A**			**726 nm laser-sample B**		
	**1.2 mW**	**6 mW**	**30 mW**	**1.2 mW**	**6 mW**	**30 mW**
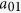	0.1129	0.0428	0.0330	0.0251	0.0179	0.0197
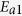 (m eV)	3.66	3.01	0.060	2.55	0.74	0.17
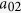	5277110	324333	19796	30675100	2370330	75011
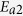 (m eV)	236.83	227.96	198.18	241.49	230.11	207.54
